# ‘Photonic Hook’ based optomechanical nanoparticle manipulator

**DOI:** 10.1038/s41598-018-20224-4

**Published:** 2018-02-01

**Authors:** Angeleene S. Ang, Alina Karabchevsky, Igor V. Minin, Oleg V. Minin, Sergey V. Sukhov, Alexander S. Shalin

**Affiliations:** 1 Electrooptical Engineering Unit, Ben-Gurion University, Beer-Sheva, 8410501 Israel; 2 Ilse Katz Institute for Nanoscale Science & Technology, Ben-Gurion University, Beer-Sheva, 8410501 Israel; 3 “Nanooptomechanics” Laboratory, ITMO University, St Petersburg, 199034 Russia; 4 Tomsk State Politechnical University, 36 Lenin Avenue, Tomsk, 634050 Russia; 5 Tomsk State University, 30 Lenin Avenue, Tomsk, 634050 Russia; 6 CREOL, The College of Optics and Photonics, University of Central Florida, Orlando, Florida 32816 USA

## Abstract

Specialized electromagnetic fields can be used for nanoparticle manipulation along a specific path, allowing enhanced transport and control over the particle’s motion. In this paper, we investigate the optical forces produced by a curved photonic jet, otherwise known as the “photonic hook”, created using an asymmetric cuboid. In our case, this cuboid is formed by appending a triangular prism to one side of a cube. A gold nanoparticle immersed in the cuboid’s transmitted field moves in a curved trajectory. This result could be used for moving nanoparticles around obstacles; hence we also consider the changes in the photonic hook’s forces when relatively large glass and gold obstacles are introduced at the region where the curved photonic jet is created. We show, that despite the obstacles, perturbing the field distribution, a particle can move around glass obstacles of a certain thickness. For larger glass slabs, the particle will be trapped stably near it. Moreover, we noticed that a partial obstruction of the photonic jet’s field using the gold obstacle results in a complete disruption of the particle’s trajectory.

## Introduction

The concept of electromagnetic radiation producing mechanical action on particles has a long history and a wide array of practical applications^1^. During the 19^th^ century, P. N. Lebedev showed the first experimental demonstration of this phenomena^2^. Using auxiliary structures such as hyperbolic metamaterials^3,4^, photonic crystals^4–6^, optical nanofibers and waveguides^7,8^, localized plasmons^9,10^ or propagating surface plasmon-polaritons^11,12^, one can create structured electromagnetic fields for generating optical forces more complex than those in simple optical traps, opening a room of opportunities for enhanced optical transport and flexible control over the motion of particles. These methods of optical manipulation have recently attracted significant attention as they pave the way to the development of complex nano-mechanical devices^13^ and optically reconfigurable nanostructures^14,15^.

It is known that dielectric microparticles with refractive index between 1 and 2 allow the creation of a so called “photonic nanojet” (PNJ), a highly-localized, subwavelength, low-divergence beam^16^. This system could be considered as a perspective auxiliary system for optical manipulation. In particular, PNJs are studied for their capability to go beyond the diffraction limit^17^, and they can be used for various applications such as biological manipulation^18,19^, high-resolution microscopy^16,17,20^, and enhancing inelastic spectroscopy^21–23^. PNJs are primarily generated using symmetric systems such as dielectric spheres, cylinders^16,24^ and much more rarely – using non-symmetrical particles^17,25^. It was also previously found that a dielectric corner^26^ and asymmetric systems can create curved PNJs^27^.

The structured field of PNJ may induce specific optical forces. In their pioneer work, Cui *et al*.^28^ calculated the forces acting on a metallic nanoparticle immersed in a PNJ; among the results they found was the reversion of the optical force for different polarizations of the incident beam. Other previous works on the topic include generating a PNJ from a sphere trapped using double co-propagating optical tweezers^29^, the forces from a PNJ near subwavelength slits^30,31^, and another study showing that the standing waves produced by a pair of elongated PNJs is suitable for trapping nanoparticles^32^. Three-dimensional optical traps using PNJs were also considered^33^. We emphasize that the previous works regarding PNJ-induced optical forces focused on trapping particles along the axis of symmetry.

In this paper, we present a curved photonic jet, “*photonic hook*” – a structured field formed by an asymmetric dielectric particle^27^ – as a method of generating optical forces for moving particles in a curved trajectory. Hence, the photonic hook field combines the features of both PNJs and Airy beams and other self-accelerating beams^34^. The forces produced by self-accelerating beams were previously studied^35,36^, however, the generation of accelerating beams is usually achieved using complex, expensive, and demanding techniques^37^ or through a cylindrical telescopic system^38^. The presence of bulky optical elements makes regular implementations of self-accelerating beams unsuitable for microfluidic applications and ‘lab-on-a-chip’ platforms. On the contrary to the usual self-accelerating beams, the photonic hook can be created with a compact microscopic optical element, glass cuboid^27^. Here, we show that this simple asymmetric cuboid works as an auxiliary structure for advanced subwavelength optical micromanipulation, as a method of particle transport along a curved trajectory for potential physical and biological applications. The opto-mechanical effects of structured field that incorporates properties of PNJs and self-accelerating beams were never studied before and in this respect our research is absolutely novel. We consider the behavior of the photonic hook and optical forces in the cases when obstacles of gold or glass with various thicknesses are introduced at the shadow side. We demonstrate that a particle could go around the glass obstacle or be stably trapped near it, which allows new applications in optical tweezing, as well as in making nanomanipulation more flexible.

## Forces from Unobstructed Photonic Hook

**Figure 1:**
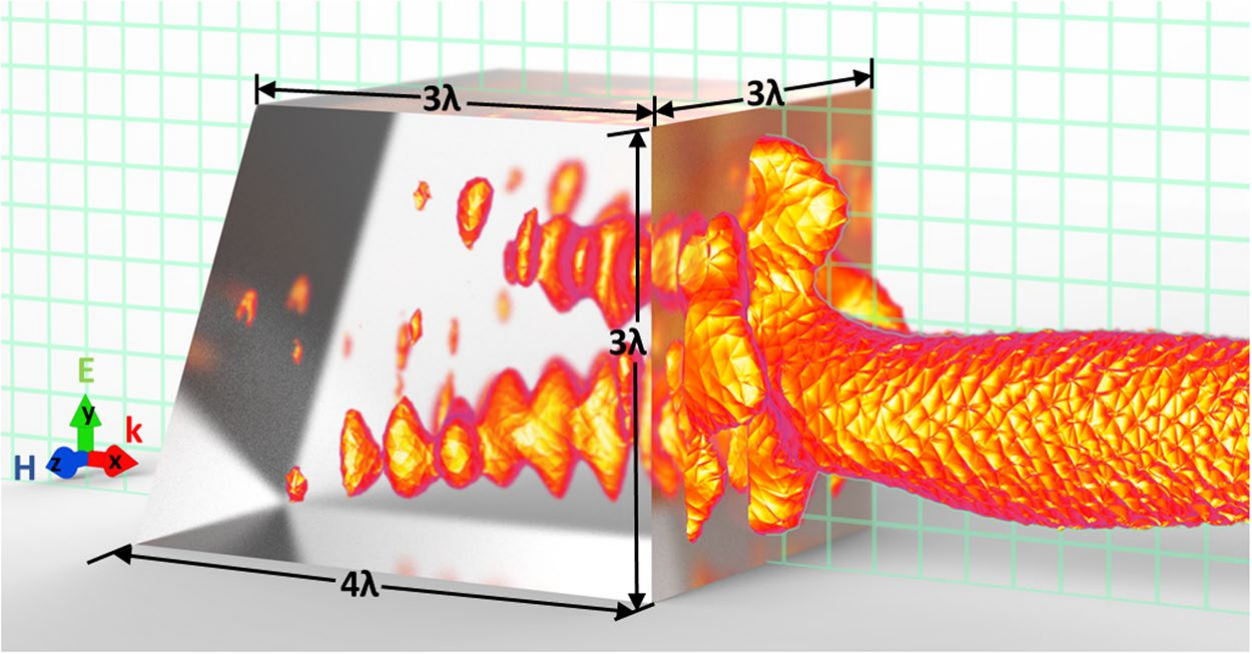
An illustration of the system and the produced photonic hook. The hook is shown as an isosurface of the amplitude of the electric field protruding from the cuboid. The grid intersecting the cuboid represents the z = 0 plane. The parameters are taken from^27^.

**Figure 2:**
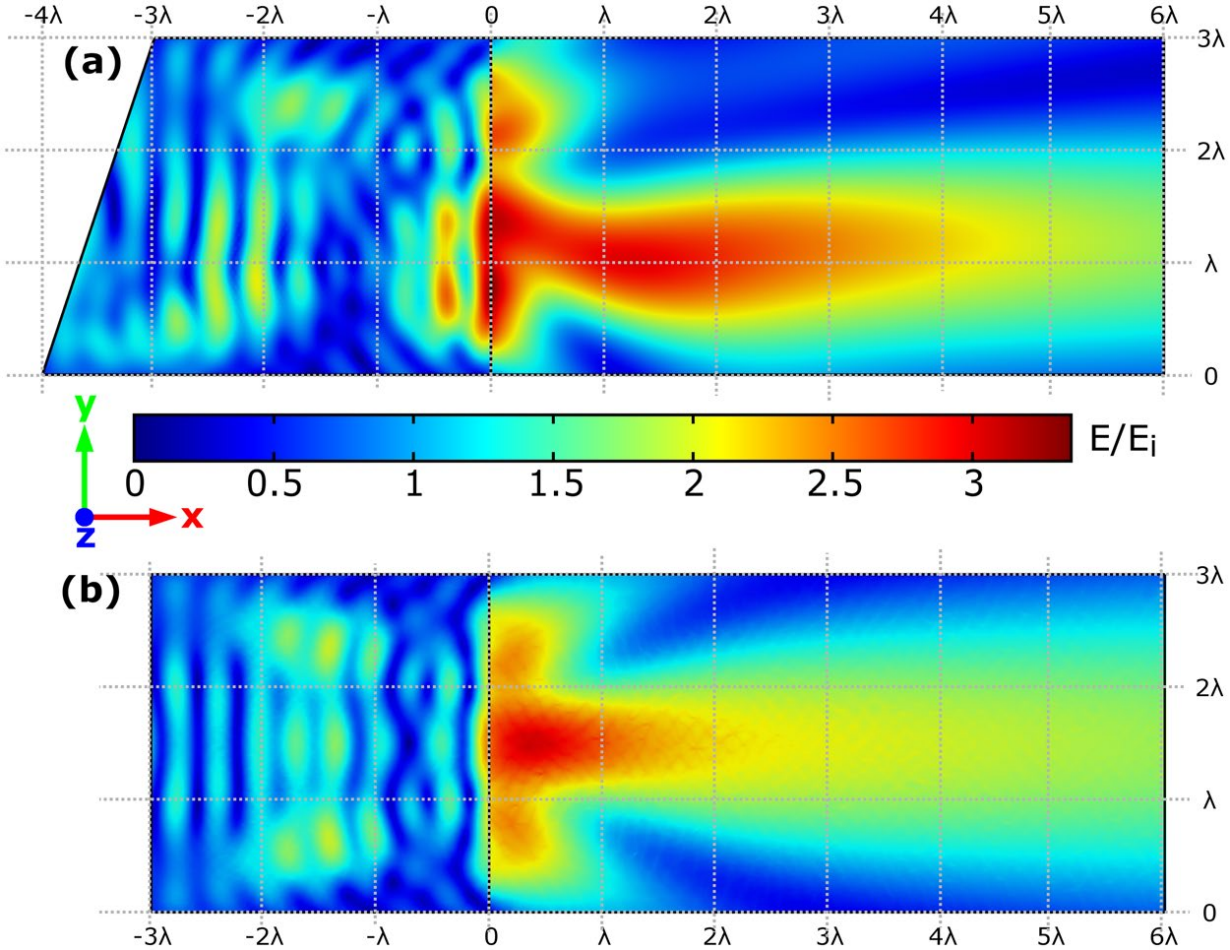
Comparison of the field strength E relative to the incident field E_i_ produced by the (**a**) asymmetric cuboid and a (**b**) cube shown in Fig. 1. The directions of the axes shown here are used for all figures throughout the paper. Parameters of the cuboid are shown in Fig. 1; The cube has edges with length of 3λ. Both figures use the same colorbar scale.

We consider the cuboid geometry shown in Fig. 1: The cuboid material has refractive index of 1.46 (which corresponds to fused silica in the visible spectral range^39^), illuminated by a plane wave, wavelength 625 nm, propagating along the x-axis, and polarized along the y-direction, embedded in air. The detailed description of the numerical simulation can be found in the Methods section. The amplitude of electric field **E** in the z = 0 plane is shown in Fig. 2a. Comparing this photonic jet to the one produced by a cube, shown in Fig. 2b, made of the same material and illuminated with the same wave, we see that the photonic nanojet becomes curved due to the wedge introduced in the system^27^. We also note that the field produced by the photonic hook has a slightly higher intensity than that of the cube.

When introducing the prism adjacent to the irradiated surface (the input) of the cube, the phase of the transmitted wave in upper and lower parts of the system changes due to the varying thickness of the prism. Accordingly, the electric field strength, illustrated in Fig. 2a, becomes non-symmetric, showing a concave deformation and this forms the curved photonic jet near the shadow surface. Thus, the symmetry breaking allows controlling the shape and the length of the photonic jet: when the triangular prism is added at the irradiated side of the cube, the photonic jet transforms into a pronounced photonic hook. A more detailed consideration of photonic hook phenomenon is described in ref.^27^. If we introduce the prism adjacent to the shadow surface (the output) of the cube, the photonic hook will not be formed^27^. Let us now consider the possibility to employ this outstanding property to achieve curved trajectory of the motion of a trapped nanoparticle, which was previously obtained with help of Airy beams only^35^. One possible application of this study is for optical fractionation^40^: a mixture of particles with differing refractive indices can be separated using their respective response to the field.

Mathematically, the optical forces acting on an arbitrary object could be obtained by integrating the Maxwell stress tensor^41,42^ over the surface, surrounding the object. However, this method requires a long computation time: to obtain the full force field, the simulations need to be performed again every time the probe particle is moved around the domain. To simplify the calculation procedure, the forces can be calculated in an approximation of the particle by an electric dipole; this method applies for Rayleigh particles (particle much smaller than the incident wavelength). The formula for the force in electric dipole approximation is^41^

1 $$\langle {{F}}_{i}\rangle =\frac{1}{2}{\rm{R}}{\rm{e}}(\alpha {\bf{E}}\cdot \frac{\partial {{\bf{E}}}^{\ast }}{\partial {{x}}_{i}}),$$

where *x*_*i*_ is the spatial coordinate (i = 1, 2, 3), ${\alpha }={\alpha }{^{\prime} }+i{\alpha }{^{\prime\prime} }$ is the particle’s complex polarizability, and **E** is the electric field acting on the particle. From this formula, the force can be written as the superposition of gradient and scattering forces^41^,

2 $$\langle {{F}}_{{i}}\rangle =\frac{\alpha ^{\prime} }{4}\frac{\partial }{\partial {x}_{i}}\langle {|{\bf{E}}|}^{2}\rangle +\frac{\alpha ^{\prime\prime} }{2}\langle {|{\bf{E}}|}^{2}\rangle \frac{\partial }{\partial {x}_{i}}\phi .$$

The first term of Eq. (2), the gradient force, is proportional to the intensity variation of the electric field; this force is responsible for particle trapping. The second term, the scattering force, arises from the momentum of photons; this non-conservative force acts in the direction of the light propagation (φ is the field phase) and can cause unrestricted particle motion.

**Figure 3:**
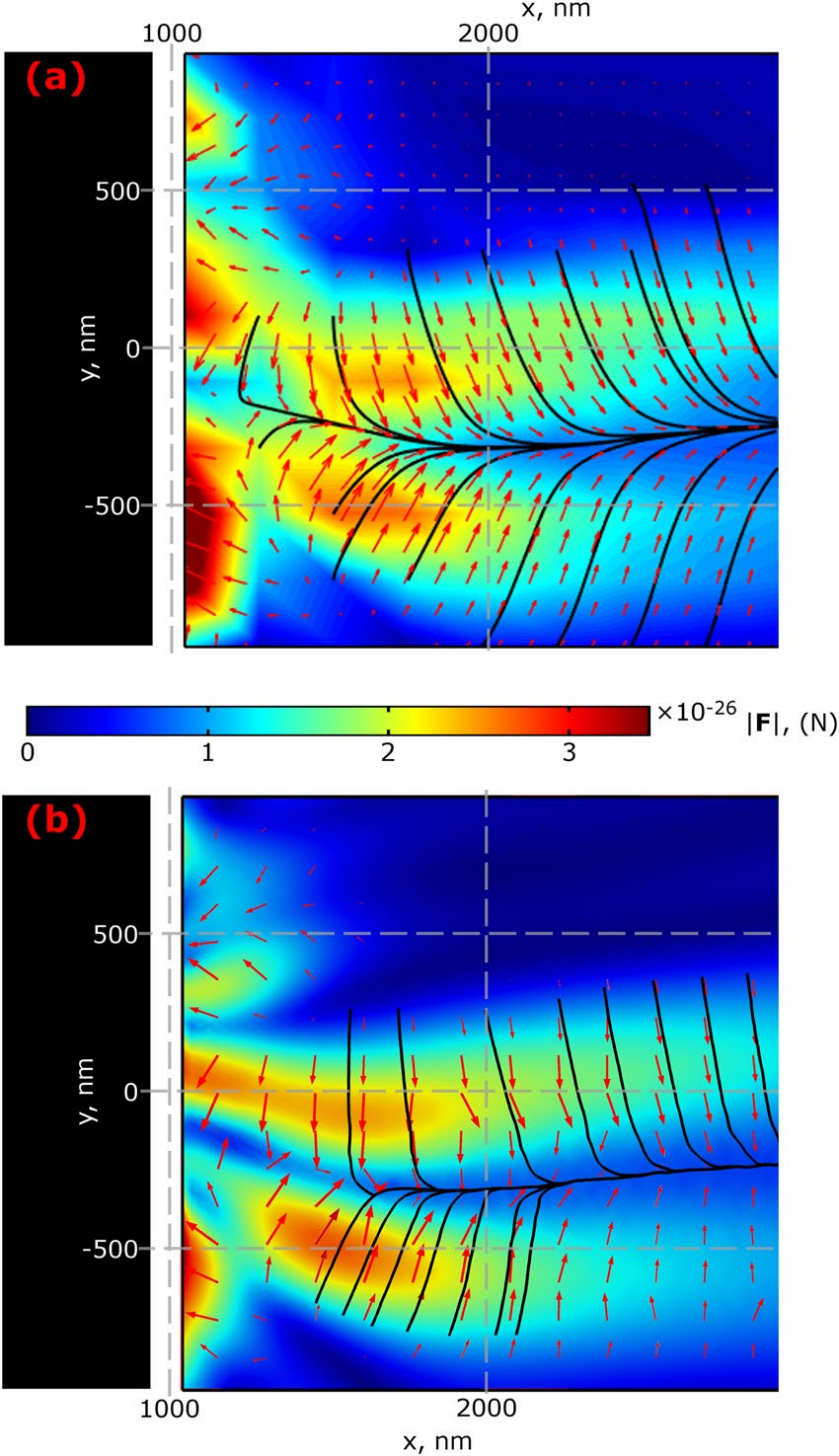
Optical forces obtained for the cuboid using MST integration (**a**) and the dipolar approximation (**b**). The color map shows the force magnitude; the streamlines and arrows represent the possible trajectories and force direction correspondingly. The black region at the left represents the location of the cuboid and the white region has the thickness equal to the particle radius. Both figures use the same colorbar scale.

First, we investigate the forces produced by the photonic hook without the presence of any obstacles, to figure out if a curved trajectory can be achieved. Here, we compare the results obtained with help of integrating the Maxwell stress tensor on a spherical surface around a gold probe particle, with size 0.03 µm and complex refractive index^43^ ϵ = −11.208 + 1.3184 *i*, shown in Fig. 3a, as well as the results, obtained using the dipolar approximation (Eq. (1)), shown in Fig. 3b.

Figure 3a and b show that the resulting forces are nearly identical, since the size of the probe particle used is within the Rayleigh regime. We also compared the forces generated by a Gaussian beam in free space with waist diameter equals to λ; the force generated by the unobstructed photonic hook is about ten times larger in magnitude than that of the Gaussian beam. Additionally, it was previously shown^4^ that the presence of the Rayleigh probe does not drastically change the field, hence we will use the semi-analytical model (numerically calculated field distributions which are used for analytical calculations of forces) for simplification in later results.

## Obstructed Photonic Hook Forces

It is well known that the introduction of a large particle, beyond the Rayleigh regime, in the path of a laser beam can introduce a disturbance in the field due to absorption and scattering. Since we aim to consider the application of photonic hook for guiding particles around obstacles, we then analyze the case of gold and glass slabs placed above the hook’s path (i.e., the obstacle does not completely block the field of the original hook and a nanoparticle is supposed to go around the obstacles). The obstacles have length of 3λ along the z-axis, length of 1.5λ along the y-axis, and the width along the x-axis is varied. In this paper, we chose widths of λ/2 and λ/4, which are 312.5 nm and 156.25 nm, respectively. The obstacles are placed at a length λ/2 away from the cuboid’s right edge, which roughly corresponds to the location of maximum curvature of the hook.

**Figure 4:**
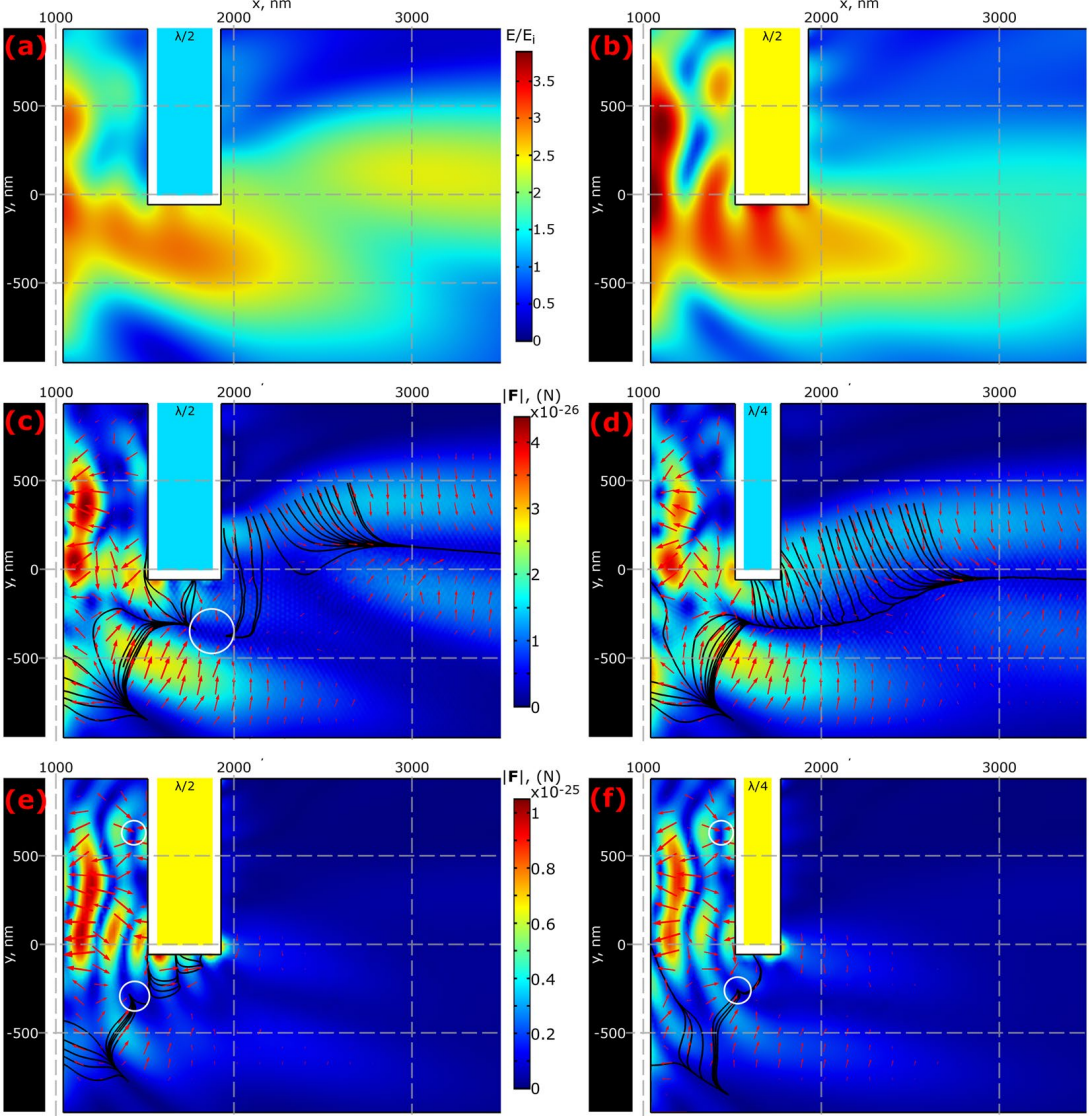
(**a**,**b**) Comparison of the field strength E relative to the incident field Ei produced when the (**a**) λ/2 glass and (**b**) λ/2 gold obstacles are introduced. (**c**–**f**) Forces produced by the cuboid system with an additional (**c**,**d**) glass obstacle with (**c**) width λ/2 and (**d**) width λ/4, and a (**e**,**f**) gold obstacle with (**e**) width λ/2 and (**f**) width λ/4. The color plot represents the force magnitude, the streamlines and arrows represent possible trajectories and the force direction, respectively. The black region at the left represents the cuboid and the white regions have thickness equal to the particle radius. White circles in (**c**), (**e**), and (**f**) show the locations of trapping. Figures (**a**) and (**b**); (**c**) and (**d**); and (**e**) and (**f**) use the same color scales.

The field perturbation, produced by the glass obstacle above the jet reveals an interesting effect: the trajectory of the particle in the region x > 2500 nm lays above the y-axis for the λ/2 case, and in the λ/4 case, the trajectory nearly coincides with the y-axis, shown in Fig. 4c and d, respectively. The phase difference produced by the field as it goes through the obstacle creates the new jet, shown in Fig. 4a. The gradient force is dominant in this case and is responsible for the trapping seen below the y-axis for the λ/2 case (trapping position is indicated with white circle in Fig. 4c); the influence of the scattering force makes the particle move away from the system in the region x >2 µm. In general, the force is attracting for the probe in the close proximity of the cuboid, but as the probe moves away from the cuboid, the particle is pushed away. Hence, in the case of the glass obstacle, the particle will move around an obstacle with λ/4 thickness, however, for a λ/2-thick glass obstacle, the particle will be trapped.

In contrast to the previous case shown in Fig. 4c and d, the introduction of the λ/4 gold obstacle to the cuboid system strongly disturbs the photonic hook, making it almost straight as can be seen in Fig. 4b. This is an interesting result because the gold obstacle is out of path of the original jet. This strong disturbance happens because the jet is produced by the wave with varying phase along the cuboid’s shadow side, so the disruption of one part of the wave leads to the disruption of the original jet (in the first case - the glass obstacle is transparent leading to the rather weak disturbance). The forces, shown in Fig. 4e for width of λ/2 and Fig. 4f for λ/4, produced by this system, create two stable trapping locations near the obstacle, as indicated by the white circles. The force pushing the particle away from the system still exists, but it is very small. The field between the cuboid and the obstacle, shown in Fig. 4b, has an appearance similar to the interference pattern of a standing wave; the forces produced by a standing wave nanojet have been previously studied^32,44^, hence we did not consider this case in our paper.

## Conclusion

To conclude, we investigated in detail the optomechanical application of the curved photonic nanojet, photonic hook, produced by an asymmetric cuboid formed by appending a triangular prism to a cube. Using numerical methods, we showed that the trajectory of a probe nanoparticle in the shadow side of the cuboid is curved. Upon the addition of the large glass obstacle to the system, the trajectory shifts towards another path, albeit losing some of its curvature in the long-range; the particle trajectory is seen bending around the glass obstacle for the λ/4 case thickness of the obstacle, whereas a stable trapping point is seen for the λ/2 case. On the other hand, upon the addition of the gold obstacle, the jet is reduced in size; the forces in the long-range are decreased as well. Additionally, stable trapping points are seen around the obstacle for both λ/4 and λ/2 thicknesses.

The revealed new optomechanical effect allows moving particles around a specific path, paving the way to enhanced and more flexible optical manipulation of nanoparticles and their transport along non-straight trajectories without the complicated employment of Airy beams. Moreover, experimental demonstrations of forces from Airy beams^35,45^ have shown results for manipulating microparticles, whereas we have shown that our method works on the scale of much smaller nanoparticles. The microscopic dimensions and simplicity of our system allows its easy integration into microfluidic devices and ‘lab-on-a-chip’ platforms. For future study, we propose embedding this cuboid on a substrate, representing a more realistic case in which this system can be used in an integrated device. The possibility to bend the particle trajectory around transparent obstacles without changing an optomechanical system opens a room of opportunities for optical, mechanical, biological research and applications, and microfluidics.

## Methods

We first simulated the cube-prism structure illustrated in Fig. 1 using Finite Element Method (FEM) software Comsol Multiphysics^46^. The ratio between the microparticle size and the wavelength of incident light is taken from ref.^27^ to obtain a pronounced photonic hook. Perfectly matched layers (PML) bound the simulation space at the all sides. At the cube’s/cuboid’s shadow side, additional space with the width 6λ is added to visualize and study the resulting photonic hook and optical forces. We used a free tetrahedral mesh with maximum element size λ/5 for the free-space regions, and λ/5/1.5 for the cuboid. Additionally, we used a free triangular mesh with maximum element size λ/20 for the z-plane to obtain figures of better resolution – the need for very fine meshes in PNJ numerical simulations is a known problem^28^, and an even finer mesh is required for obtaining the field’s spatial derivatives to obtain the forces.

For the all force maps presented, we introduced a gold sphere with radius 0.03 µm as a probe particle. The incident wavelength is set to 625 nm (f = 480 THz), which corresponds to red visible light. At this frequency, the probe has dielectric permittivity^43^ ϵ = −11.208 + 1.3184 i. For the simulation used to produce Fig. 3a, the forces were evaluated in a 10 × 10 rectangular grid behind the cuboid (shadow side), and the results were interpolated and plotted using Matlab.

## References

[1] Wördemann, M. *Structured Light Fields: Applications in Optical Trapping, Manipulation, and Organisation*. (Springer Science & Business Media, 2012).

[2] Lebedev P (1901). Untersuchungen über die Druckkräfte des Lichtes. Ann. Phys..

[3] Shalin, A. S., Sukhov, S. V., Bogdanov, A. A., Belov, P. A. & Ginzburg, P. Optical pulling forces in hyperbolic metamaterials. *Phys. Rev. A* **91** (2015).

[4] Bogdanov AA, Shalin AS, Ginzburg P (2015). Optical forces in nanorod metamaterial. Sci. Rep..

[5] Ang, A. S., Sukhov, S. V., Dogariu, A. & Shalin, A. S. Scattering forces within a left-handed photonic crystal. *Sci. Rep.* **7**, 41014 (2017). 10.1038/srep41014 PMC5253622 28112217

[6] Benabid F, Knight JC, Russell PSJ (2002). Particle levitation and guidance in hollow-core photonic crystal fiber. Opt. Express.

[7] Xin H, Cheng C, Li B (2013). Trapping and delivery of Escherichia coli in a microfluidic channel using an optical nanofiber. Nanoscale.

[8] Shalin AS, Ginzburg P, Belov PA, Kivshar YS, Zayats AV (2014). Nano-opto-mechanical effects in plasmonic waveguides. Laser Photonics Rev..

[9] Juan ML, Righini M, Quidant R (2011). Plasmon nano-optical tweezers. Nat. Photonics.

[10] Shalin AS, Sukhov SV (2012). Optical forces in plasmonic nanoantennas. Quantum Electron..

[11] Shalin, A. S. & Sukhov, S. V. Plasmonic nanostructures as accelerators for nanoparticles: Optical nanocannon. *Plasmonics* **8**, 625–629 (2013).

[12] Ivinskaya A (2016). Plasmon-assisted optical trapping and anti-trapping. *Light Sci*. Appl..

[13] Rahneshin, V., Khosravi, F., Ziolkowska, D. A., Jasinski, J. B. & Panchapakesan, B. Chromatic mechanical response in 2-D layered transition metal dichalcogenide (TMDs) based nanocomposites. *Sci. Rep.* **6**, srep34831 (2016). 10.1038/srep34831 PMC5054383 27713550

[14] Liu, M., Powell, D. A., Guo, R., Shadrivov, I. V. & Kivshar, Y. S. Polarization-induced chirality in metamaterials via optomechanical interaction. *Adv. Opt. Mater*. **5**, n/a-n/a (2017).

[15] Shadrivov IV, Kapitanova PV, Maslovski SI, Kivshar YS (2012). Metamaterials Controlled with Light. Phys. Rev. Lett..

[16] Chen Z, Taflove A, Backman V (2004). Photonic nanojet enhancement of backscattering of light by nanoparticles: a potential novel visible-light ultramicroscopy technique. Opt. Express.

[17] Luk’yanchuk BS, Paniagua-Domínguez R, Minin IV, Minin OV, Wang Z (2017). Refractive index less than two: photonic nanojets yesterday, today and tomorrow [Invited]. Opt. Mater. Express.

[18] Li Y-C (2016). Manipulation and detection of single nanoparticles and biomolecules by a photonic nanojet. *Light Sci*. Appl..

[19] Li Y (2016). Trapping and Detection of Nanoparticles and Cells Using a Parallel Photonic Nanojet Array. ACS Nano.

[20] Wang, Z. *et al*. Optical virtual imaging at 50 nm lateral resolution with a white-light nanoscope. *Nat. Commun*. **2**, ncomms1211 (2011). 10.1038/ncomms1211 21364557

[21] Kasim J (2008). Near-field Raman imaging using optically trapped dielectric microsphere. Opt. Express.

[22] Yi KJ, Wang H, Lu YF, Yang ZY (2007). Enhanced Raman scattering by self-assembled silica spherical microparticles. J. Appl. Phys..

[23] Lecler S (2007). Photonic jet driven non-linear optics: example of two-photon fluorescence enhancement by dielectric microspheres. Opt. Express.

[24] Zhao L, Ong CK (2009). Direct observation of photonic jets and corresponding backscattering enhancement at microwave frequencies. J. Appl. Phys..

[25] Minin IV, Minin OV, Geints YE (2015). Localized EM and photonic jets from non-spherical and non-symmetrical dielectric mesoscale objects: Brief review. Ann. Phys..

[26] Kotlyar VV, Stafeev SS, Kovalev AA (2013). Curved laser microjet in near field. Appl. Opt..

[27] Minin, I. V. & Minin, O. V. *Diffractive Optics and Nanophotonics*. (Springer International Publishing), 10.1007/978-3-319-24253-8 (2016).

[28] Cui X, Erni D, Hafner C (2008). Optical forces on metallic nanoparticles induced by a photonic nanojet. Opt. Express.

[29] Neves AAR (2015). Photonic nanojets in optical tweezers. J. Quant. Spectrosc. Radiat. Transf..

[30] Valdivia-Valero FJ, Nieto-Vesperinas M (2012). Optical forces on cylinders near subwavelength slits: effects of extraordinary transmission and excitation of Mie resonances. Opt. Express.

[31] Valdivia-Valero FJ, Nieto-Vesperinas M (2013). Optical forces on cylinders near subwavelength slits illuminated by a photonic nanojet. Opt. Commun..

[32] Wang H, Wu X, Shen D (2016). Trapping and manipulating nanoparticles in photonic nanojets. Opt. Lett..

[33] Yannopapas V (2012). Photonic nanojets as three-dimensional optical atom traps: A theoretical study. Opt. Commun..

[34] Mathis A (2013). Arbitrary nonparaxial accelerating periodic beams and spherical shaping of light. Opt. Lett..

[35] Baumgartl J, Mazilu M, Dholakia K (2008). Optically mediated particle clearing using Airy wavepackets. Nat. Photonics.

[36] Schley, R. *et al*. Loss-proof self-accelerating beams and their use in non-paraxial manipulation of particles’ trajectories. *Nat. Commun*. **5**, ncomms6189 (2014). 10.1038/ncomms6189 25355605

[37] Siviloglou, G. A., Broky, J., Dogariu, A. & Christodoulides, D. N. Observation of accelerating Airy beams. *Phys. Rev. Lett.* **99**, 213901 (2007). 10.1103/PhysRevLett.99.213901 18233219

[38] Papazoglou DG, Suntsov S, Abdollahpour D, Tzortzakis S (2010). Tunable intense Airy beams and tailored femtosecond laser filaments. Phys. Rev. A.

[39] Malitson IH (1965). Interspecimen Comparison of the Refractive Index of Fused Silica. JOSA.

[40] MacDonald MP, Spalding GC, Dholakia K (2003). Microfluidic sorting in an optical lattice. Nature.

[41] Novotny, L. & Hecht, B. *Principles of Nano-Optics*. (Cambridge University Press, 2006).

[42] Gao D (2017). Optical manipulation from the microscale to the nanoscale: fundamentals, advances and prospects. Light Sci. Appl..

[43] Johnson PB, Christy RW (1972). Optical constants of the noble metals. Phys. Rev. B.

[44] Minin IV, Minin OV, Pacheco-Peña V, Beruete M (2016). Subwavelength, standing-wave optical trap based on photonic jets. Quantum Electron..

[45] Zheng Z, Zhang B-F, Chen H, Ding J, Wang H-T (2011). Optical trapping with focused Airy beams. Appl. Opt..

[46] COMSOL Multiphysics. Available at: https://www.comsol.com/. (Accessed: 22nd August 2017).

